# Gender Differences in Sexual Behaviors and Their Relevance to Mental Health among High School Students with Sexual Experience in South Korea

**DOI:** 10.3390/ijerph182111295

**Published:** 2021-10-27

**Authors:** Hyunlye Kim, Kwang-Hi Park, Suin Park

**Affiliations:** 1 Department of Nursing, College of Medicine, Chosun University, Gwangju 61452, Korea; hlkim5207@chosun.ac.kr; 2 College of Nursing, Gachon University, Incheon 21936, Korea; parkkh@gachon.ac.kr; 3 College of Nursing, Kosin University, Busan 49267, Korea

**Keywords:** adolescent, gender differences, sexual behavior, mental health, suicide

## Abstract

We explored gender differences in sexual behavior, and their relevance to mental health among high school students in South Korea. This study was based on data from the 14th Korea Youth Risk Behavior Web-Based Survey (2018). The subjects were 2460 high school students who reported sexual experiences. Student′s *t*-test, ANOVA, and Rao-Scott chi-squared test were performed to identify the significance of the differences. Boys had earlier sexual debuts (Rao-Scott χ^2^ = 53.55, *p* < 0.001), a lower frequency of using contraceptives (Rao-Scott χ^2^ = 26.57, *p* < 0.001), and lower rates of sex education in school (Rao-Scott χ^2^ = 11.20, *p* = 0.004). With respect to mental health factors, there was a difference in suicidality according to sexual risk behaviors, with a stronger association found in boys. In girls, there was an association between pregnancy experiences and suicidal ideation (Rao-Scott χ^2^ = 9.90, *p* = 0.003), plans (Rao-Scott χ^2^ = 17.25, *p* < 0.001), and attempts (Rao-Scott χ^2^ = 23.11, *p* < 0.001). Our findings suggest differences by gender and age group in the association between sexual behavior and mental health. It is necessary to devise a sex education strategy for adolescents considering gender and early versus late adolescent development period.

## 1. Introduction

Adolescence, derived from the Latin word *adolescere*, meaning ‘to grow’, is an important period of development [1]. Sexual development in adolescence is a complex biological and psychosocial process, which Kar and colleagues [1] have referred to as ‘a bumpy ride’. Teenage sexuality is often seen as dangerous, and the contradictory and biased views of adults can hamper adolescents’ ability to negotiate the difficult realms of desire and risk [2]. Studies of sexuality in adolescence and early adulthood tend to focus on romantic partnerships as a developmental task, rather than exploring their relationships with potential public health issues and other risky behaviors, such as sexually transmitted diseases and unwanted pregnancies [3]. This study intends to explore the sexual behavior of high school students in South Korea from a broader perspective in consideration of this trend regarding adolescent sexuality, using data from a nationally representative sample of adolescents.

Sexual activity can vary significantly between early and late adolescence. According to a health behavior survey of approximately 15% of all South Korean adolescents, the rate of sexual intercourse experience was 2.6% for middle school students and 8.5% for high school students [4]. In a previous study in Ethiopia, 29.8% of high school students who participated in the study had sexual experience, and 64.8% of them had their first sex under the age of 18 [5]. Vasilenko, Lefkowitz, and Welsh [6] reported that early sexual behavior may be associated with negative health outcomes but could also be a positive experience in later adolescence. A study that investigated the differences in sexual knowledge and sex education needs of elementary, middle, and high school students in South Korea emphasized the need for customized sex education considering developmental characteristics [7]. That is, the higher the school level, the greater the experience of sex education, but the number of students who answered that school sex education was helpful decreased as the school level increased [7]. Regarding the content of sex education that students most wanted to learn, ‘how to deal with each situation’ was the highest across age groups, followed by the youth protection law in middle school students, and the contraceptive method in high school students [7]. By focusing on high school students in late adolescence who have sexual experience, we intend to suggest a direction for sex education for them.

Gender differences in sexuality have long been a research target. A 1993–2007 meta-analysis of gender differences in sexuality found that men typically reported more sexual experiences and more tolerant attitudes than women [8]. These differences are also apparent in adolescence, and adolescent sexuality and sexual behavior also differ according to gender [9]. The gender double-standard influences sexual development and gender discrimination [10]. That is, whereas social norms encourage girls to refrain from having sex and avoid multiple sexual partners, boys are generally more sexually uninhibited because of exposure to a peer environment characterized by greater acceptance of sex and greater pressure to engage in sexual activity [3]. In a study of gender differences in sexual behavior among a large group of adolescent students in Spain, the prevalence of risky sexual behaviors (e.g., multiple sex partners, non-use of contraceptives) was generally higher in boys than girls [11]. However, this research approach to gender differences in adolescent sexuality is now outdated.

Adolescent sexuality is significantly related to health outcomes. Although the formation of romantic relationships and participation in sexual behavior are currently considered normative and important developmental tasks for adolescents and young adults [12], there is evidence of an association between early sexual experience and health status. Vasilenko and colleagues [6] proposed a conceptual model of the effects of adolescent sexual behavior on physical, mental, and social health. For example, physically, adolescents who are sexually active are also more likely to acquire and transmit sexually transmitted infections [13]. In terms of mental health, an association between suicidal tendencies and risky sexual behaviors, such as early sexual intercourse and contraceptive use, was reported in a South Korean adolescent population [14,15]. A study of high school students in the United States reported associations of stress and depression with sexually risky behaviors [16]. Another mental health study found a positive association between sexual intercourse and suicide attempts among adolescents in 32 of 38 countries across the four World Health Organization (WHO) regions [17]. “Sexting” (the sharing of sexually suggestive photos) may be a gateway behavior to early sexual activity and may also increase the likelihood of social ostracism [18]. The rate of consensual sexting was significantly higher among students who reported depressive symptoms, cyberbullying, and suicide attempts [18]. Although mental health has been shown to be related to adolescent sexual behavior, more research on this association is required. Therefore, we aimed to explore gender differences in sexual behavior, and their relevance to mental health among high school students with sexual experience in South Korea. This study defined the following two hypotheses: (1) There will be gender differences in the sexual behavior of high school students; (2) The relationship between sexual behavior and mental health will be different for boys and girls in high school.

## 2. Materials and Methods

### 2.1. Design and Data Source

This secondary analysis was based on the 14th Korea Youth Risk Behavior Web-Based Survey (KYRBS), 2018 [4], conducted by the Ministry of Education, Ministry of Health and Welfare, and Centers for Disease Control and Prevention (now known as the Korea Disease Control and Prevention Agency, KDCA). The KYRBS is an ongoing survey which was initiated in 2005 to monitor health behaviors among middle and high school students nationwide. We analyzed the data from the survey conducted from September to November 2018. The 2005 KYRBS questionnaire (anonymous and self-administered) included 92 items in 11 domains and was expanded to 103 items in 15 domains in 2018. The KYRBS data can be used by researchers in accordance with the regulations on disclosure and management of raw data of the KDCA.

### 2.2. Samples

The target population of the KYRBS is South Korean middle and high school students. To obtain a representative sample, a complex multi-step sampling method including stratification, clustering, and weighting was used. To minimize sampling error, the population was divided into 117 strata based on 39 regions and three school levels (middle school, general high school, and specialized high school). The sample of 400 middle schools and 400 high schools was refined to five of each for each of the 17 cities/provinces. By applying a proportional distribution method, the population distribution was consistent according to the stratification variables. Schools and classes were randomly selected according to the stratified sampling method. All students in each selected class were surveyed. Long-term absentees, special-needs children, and students with low literacy were excluded from the sample. Of a total of 60,040 respondents (response rate = 95.6%), the data from 2460 high school students (1589 boys and 871 girls) who reported sexual experiences were analyzed in this study.

Among the 2460 respondents, 67.7% (*n* = 1589) were boys and 32.3% (*n* = 871) were girls. The mean age was 16.80 years (SE = 0.02; range 12–18 years). Students in the 12th grade accounted for the highest proportion (48.9%), followed by 11th (31.9%) and 10th graders (19.2%). The highest number of students who responded had moderate academic achievement (23.8%), and a moderate economic status (38.3%), among five response options (“high”, “medium-high”, “medium”, “medium-low”, and “low”). Most of the students (88.7%) were living with their family.

### 2.3. Measures

#### 2.3.1. Sexual Behavior

Variables related to sexual behavior analyzed in this study included time of sexual debut, sexual intercourse after drinking, contraception use, contraception method, sex education in school, and pregnancy experience. Time of sexual debut was evaluated by the question: “When was the first time you had sex?”. The original response options ranged from “before entering elementary school” (1) to “third year of high school” (13). These options were recategorized to three options (“elementary school or before”, “middle school”, and “high school”). The question on sexual intercourse after drinking was: “Have you ever had sex after drinking alcohol?”, with “yes” and “no” as the response options. Contraception use during sexual intercourse was assessed by the question: “Did you use birth control during sexual intercourse to prevent pregnancy?”, with response options ranging from “not at all” (1) to “always” (4). Another question on contraception was as follows: “What is your main method of contraception? (choose one main method)”. The response options were “oral contraceptive oral pill”, “condom”, “extravaginal ejaculation” and “other” (including menstrual cycle method, emergency contraceptive pill, and intrauterine device). The question regarding sex education in school was: “In the past 12 months, have you received any sex education at school (including classes, broadcasts, or lectures in auditoriums)?”; “yes” and “no” were the response options. The question on pregnancy experience, which was only answered by the girls, was assessed by the question: “Have you ever been pregnant?”, with “yes” and “no” as the response options.

#### 2.3.2. Mental Health

The mental health areas of interest were perceived stress, depressed mood, and suicidal ideation/plans/attempts. Perceived stress was evaluated by the question: “What level of stress do you usually feel?” The original response options ranged from “very high” (1) to “none at all” (5). In this study, perceived stress was recorded in reverse, so higher scores indicate higher stress. Depressed mood was assessed by the yes/no question: “In the past 12 months, have you ever felt so sad or hopeless that you stopped your daily activities for 2 full weeks?”. Suicidal ideation/plans/attempts during the last 12 months were evaluated by yes/no questions: “In the past 12 months, have you seriously considered suicide in the past 12 months?”, “In the past 12 months, have you made any concrete plans to commit suicide in the last 12 months?”, and “In the past 12 months, have you attempted suicide in the last 12 months?”.

### 2.4. Data Analyses

The data were analyzed using IBM SPSS Statistics (version 24.0; IBM Corp., Armonk, NY, USA) with adjustment for the complex sampling design. We conducted a frequency analysis to obtain weighted percentages and standard error (SE). Rao-Scott χ^2^ test was performed to identify differences in sexual behavior by gender. Student′s *t*-test, ANOVA, and Rao-Scott χ^2^ test were performed to identify differences in mental health according to sexual behavior. Significance was set at *p* < 0.05 (two-sided).

## 3. Results

### 3.1. Differences in Sexual Behavior by Gender

Table 1 shows the differences in sexual behavior by gender. More boys than girls had their first sexual intercourse when in elementary school or before (boys, 17.4%; girls, 8.6%) or when in middle school (boys, 37.2%; girls, 32.5%) (Rao-Scott χ^2^ = 53.55, *p* < 0.001). The proportion of non-use of contraception was higher in boys (29.0%) than girls (20.2%) (Rao-Scott χ^2^ = 26.57, *p* < 0.001). Among the respondents who had ever used contraception, both boys and girls used condoms the most as a contraceptive method, with more frequency of condom use in boys (83.4%) than girls (76.2%) (Rao-Scott χ^2^ = 14.35, *p* = 0.012). More girls received sex education (77.4%) than boys (71.1%) in the last year (Rao-Scott χ^2^ = 11.20, *p* = 0.004). There was no significant difference between boys and girls in the rate of sexual intercourse after drinking. Finally, 6.7% (girls only) had pregnancy experience.

### 3.2. Differences in Mental Health According to Sexual Behavior in Boys

Table 2 shows the differences in mental health according to sexual behavior in boys. The frequency of feeling sad or hopeless enough to stop daily activities for 2 full weeks in the last year was higher in those reporting having had sexual intercourse after drinking (42.5%, Rao-Scott χ^2^ = 10.51, *p* = 0.001), and was the highest in the “others” category of contraception method (59.5%, Rao-Scott χ^2^ = 11.03, *p* = 0.027). The rate of respondents with suicidal ideation in the last year was higher in those reporting having sexual intercourse after drinking (21.5%, Rao-Scott χ^2^ = 6.85, *p* = 0.013). Both the proportion of respondents with suicide plans and suicide attempts in the last year were the highest in respondents whose sexual debut time was elementary school or before (15.3%, Rao-Scott χ^2^ = 27.44, *p* < 0.001; 13.0%, Rao-Scott χ^2^ = 24.85, *p* < 0.001, respectively) and was higher amongst those reporting having sexual intercourse after drinking (11.3%, Rao-Scott χ^2^ = 20.19, *p* < 0.001; 9.6%, Rao-Scott χ^2^ = 21.53, *p* < 0.001, respectively). The rate of suicide plans and suicide attempts were the highest in respondents who reported that they sometimes use contraception (12.2% and 9.4%, respectively), followed by no contraception use (9.6%, Rao-Scott χ^2^ = 16.43, *p* = 0.005; 8.7%, Rao-Scott χ^2^ = 18.56, *p* = 0.003, respectively). The rate of suicide plans and suicide attempts was the highest in the “others” category of contraception method (39.8%, Rao-Scott χ^2^ = 38.22, *p* < 0.001; 26.7%, Rao-Scott χ^2^ = 30.54, *p* < 0.001, respectively). There was no significant association between sexual-behavior-related variables and perceived stress.

### 3.3. Differences in Mental Health according to Sexual Behavior in Girls

Table 3 shows the differences in mental health according to sexual behavior in girls. The level of perceived stress was higher in girls reporting no pregnancy experience (weighted mean = 3.78, SE = 0.03, Wald *F* = 7.47, *p* = 0.007). The proportion of suicidal ideation in the last year was higher in respondents who had pregnancy experience (46.8%, Rao-Scott χ^2^ = 9.90, *p* = 0.003). The frequency of suicide plans in the last year was the highest in respondents whose sexual debut time was elementary school or before (30.5%, Rao-Scott χ^2^ = 15.30, *p* < 0.001), and was higher in respondents who had pregnancy experience (34.2%, Rao-Scott χ^2^ = 17.25, *p* < 0.001). The proportion of reported suicide attempts in the last year was the highest in respondents whose sexual debut time was elementary school or before (27.7%, Rao-Scott χ^2^ = 15.72, *p* < 0.001), and was higher for those reporting having sexual intercourse after drinking (14.9%, Rao-Scott χ^2^ = 4.20, *p* = 0.033). The proportion of suicide attempts was also the highest in those reporting no contraception use (16.9%, Rao-Scott χ^2^ = 10.62, *p* = 0.013), and was higher in respondents who had pregnancy experience (35.1%, Rao-Scott χ^2^ = 23.11, *p* < 0.001). There was no significant association between sexual-behavior-related variables and depression.

## 4. Discussion

Sexual behavior, as a developmental task for older adolescents, is a well-established social and individual process from an ecological perspective [12]. This study explored gender differences in the relationship between sexual behavior and mental health among high school students (grades 10–12) in South Korea, which has a strong Confucian culture. In a 2018 representative survey of the Korean youth population (*N* = 29811), the sexual experience rate of high school students was 8.5% (middle school students, 2.6%), and the prevalence was 6.5–8.5% during 2007–2018 [4]. Among 12 to 15-year-olds (*N* = 116820) from 38 countries in the four WHO regions, the prevalence of sexual intercourse was 13.2% [17]. In a study including a large sample of Spanish adolescents with a mean age of 15 years (*N* = 9340), 38.7% had had sex at least once [11]. The sexual experience rate of third-year middle school students in South Korea, who are of similar age, was 4.8% [4]. As such, there are large differences in sexual characteristics between countries and cultures, so it is necessary to consider the sociocultural background to understand youth sexuality.

In this study, boys were not only more likely to have sexual experience (11.0% vs. 5.7% in girls), but also had an earlier sexual debut, were less likely to use contraceptives, and had lower rates of sex education in school compared to girls. The above-mentioned Spanish study also reported higher rates of sexual intercourse and engagement in risky behavior in boys [11]. A study exploring adolescent sexual debuts using longitudinal social network data found that girls who reported having sex experienced a significant decrease in peer acceptance over time, whereas male adolescents reporting the same behavior reported significant increases in peer acceptance [10]. In another study of sexting in adolescents, boys were more likely to report consensual sexting, and high school boys who reported sexting were more likely to engage in other risky behaviors [18]. These gender differences in adolescent sexual activity appear to stem from the gender double-standard mentioned previously. The sexual double-standard, which is reflected in sexual humility in girls and sexual profligacy in boys, can have negative effects on sexual and mental health [19]. Therefore, it is necessary to help boys and girls understand and pursue gender equality as it pertains to sexuality.

Other explanations of the riskier sexual behaviors of boys have also been provided. A study of a group of arrested adolescents at high risk of sexual risk found that optimism about the future predicted condom use and higher impulse control and was associated with a lower likelihood of casual sex [20]. As in this study, the lack of school-based sex education for boys may also be a factor in their higher rates of sexual activity. According to a study of the relationship between school-based sex education and sexual health in adolescents in four developed countries (The Netherlands, France, Australia, and the United States), adolescent sexual health is best promoted by recognizing, accepting, and appropriately regulating mutual sexuality, rather than through abstinence-based policies [21]. In other words, liberal policies do not necessarily promote sexual behavior, and it is important to ensure that young people are equipped with the skills required for behaviors that maintain sexual health. Therefore, public education and health officials need to design and provide multi-faceted sex education programs that consider the characteristics of adolescent sexuality in their culture, especially for boys.

Our analysis of the relationship between sexual behavior and suicidality according to gender showed significant results. In both genders, risky or low-responsibility sexual behaviors (early sexual debut, sexual intercourse after drinking, not using contraception, or having a partner using contraceptives rather than themselves) were associated with higher levels of suicide-related characteristics. In addition, relatively more significant associations were found in boys, and an additional association with pregnancy experience was found in girls. Irrespective of gender, an association between sexually risky behavior and suicidal ideation/plan/attempt has been reported consistently [14,15,17,22]. A stronger association between sexually risky behaviors and negative mental health among high school boys was also found in a study by Frankel et al. (2018) [18], which could be attributed to their tendency to take part in more open and risk-taking sexual behaviors [23]. As for the sample considered in the present study, an association between pregnancy experience and suicidal outcome in girls has also been found in previous studies [15,22,24]. Adolescent pregnancy remains a major public health problem worldwide, and girls experiencing this may be at a higher risk of various mental disorders and suicidality [25]. Therefore, to address adolescents’ sexual and mental health needs simultaneously, it would be useful to monitor the sexual risk behaviors of boys, and contraception use and pregnancy in girls.

In this study, some sexually risky behaviors were associated with depression, while most sexual behaviors were not associated with usual stress. These findings are somewhat different from the results of previous studies investigating the relationship between adolescent sexual behavior and mental health [16]. A study using the same data source as this study reported that adolescents with sexual experience had higher levels of stress, depression, suicidal ideation, and lower happiness [26]. In this study, the results for girls with pregnancy experience showing lower reported stress are interpreted as distorted results due to the small sample size of the analysis group (*n* = 54), the time difference between pregnancy and the reported stressful experience, and the excessive action of various life stressors other than pregnancy. Therefore, repeated studies with a larger number of samples and a more rigorous design are required to confirm such inconsistent results in the future.

This unexpected discrepancy could be explained by the difference between early and late adolescence [14,15]. Kim and Kim [14] found that the association between early sexual intercourse and mental health was strongest in preteens. In this connection, it has been proposed that understanding sexuality in late adolescence is one of the main developmental tasks related to sexual well-being and successful romantic relationships [3,6]. These interpretations suggest that to fully understand the sexual behavior of later adolescents and to improve their sexual health, that they should be treated as distinct from early adolescents. Harden [27] suggested that sexual intercourse during adolescence does not generally worsen psychological functioning; a “gender-positive framework” was proposed in contrast to the previously dominant risk-based view of adolescent sexuality. To achieve sexual well-being, the importance of understanding the psychological variables modulating sexual experience was also emphasized. The data suggest that this approach may be suitable, for later adolescents at least, in South Korea. Recent surveys of sexual activity among Korean adolescents reported relatively low rates of sexual experience (4.3–5.7% in 2003–2018), and an increasing rate of contraceptive use (39% in 2003 and 2013; 59.3% in 2018) [4]. In this study, sex education at school did not show any significant association with any mental health variables. Considering these data, appropriate sex education is required in early and late adolescence. In the case of later adolescents beginning to engage in sexual activity, helping them to build mutually respectful relationships and achieve sexual well-being is important. It is also important to protect early adolescents against the dangers of early sexual intercourse.

This study is relevant in that we have evaluated trends in sexuality according to gender and age (i.e., the early vs. late adolescent period). However, there is a possibility of response bias due to the use of web-based self-reported data. There are also limitations related to the secondary analysis, which did not use the verified mental health questionnaire and did not provide psychometric information from the original data source. We are planning an exploratory qualitative study of sexual attitudes and experiences among later adolescents to enhance understanding of their sexual behavior. Intervention studies aimed at developing sexual health programs that consider gender and the adolescent period (early vs. late) are also required.

## 5. Conclusions

In this study of a group of South Korean adolescents, sexual behaviors and their relevance to mental health differed according to gender. The boys displayed more sexually risky behaviors, and the association of these with mental health appeared stronger. The girls were found to show associations between pregnancy experience and suicide-related characteristics. For both genders, sexual risk behavior showed a significant link with more long-term and fatal suicidality, while its link with usual stress and depression was absent or low. The results of this study suggest the necessity of sex education that considers gender and the detailed developmental period of adolescents. Boys should be more protected from early and risky sexual behavior, and girls from possible pregnancy, and should be helped to find appropriate coping strategies for each situation. For later adolescents, it is necessary to respect individual choices in the sexual development process and to help them develop safer and healthier sexual behaviors.

## Figures and Tables

**Table 1:** Differences in sexual behavior by gender (*n* = 2460).

Variable	Boys (*n* = 1589, 67.7% *)		Girls (*n* = 871, 32.3% *)		Rao-Scott χ^2^ (*p*)
	*n*	% *	*n*	% *	
Time of sexual debut ^†^					
Elementary school or before	276	17.4	70	8.6	53.55 (<0.001)
Middle school	579	37.2	274	32.5	
High school	704	45.4	509	58.9	
Sexual intercourse after drinking					
No	1023	64.5	540	62.6	0.79 (0.391)
Yes	566	35.5	331	37.4	
Contraception use					
No	451	29.0	175	20.2	26.57 (<0.001)
Sometimes	167	11.3	110	13.0	
Mostly	235	14.0	169	19.0	
Always	736	45.7	417	47.7	
Contraception method ^‡^					
Oral contraceptive pill	45	4.1	35	5.3	14.35 (0.012)
Condom	951	83.4	543	76.2	
Extravaginal ejaculation	110	9.5	97	14.8	
Other ^††^	32	3.0	21	3.6	
Sex education in school ^§^					
No	439	28.9	197	22.6	11.20 (0.004)
Yes	1150	71.1	674	77.4	
Pregnancy experience ^||^					
No	-	-	816	93.3	-
Yes	-	-	54	6.7	

* Weighted; ^†^ excluded missing values; ^‡^ among respondents who had ever used contraception; ^††^ This category included menstrual cycle method, emergency contraceptive pill, and intrauterine device; ^§^ in the last year; ^||^ female students only.

**Table 2:** Differences in mental health according to sexual behavior in boys (*n* = 1589).

Variables	Perceived Stress		Depression		Suicidal Ideation		Suicidal Plans		Suicidal Attempts	
	M ^*^ (SE)	Wald F (*p*)	% *	Rao-Scott χ^2^ (*p*)	% *	Rao-Scott χ^2^ (*p*)	% *	Rao-Scott χ^2^ (*p*)	% *	Rao-Scott χ^2^ (*p*)
Sexual debut time ^†^										
Elementary school or before	3.27 (0.07)	0.20 (0.819)	38.8	0.45 (0.808)	22.6	4.22 (0.150)	15.3	27.44 (<0.001)	13.0	24.85 (<0.001)
Middle school	3.31 (0.04)		36.5		17.2		4.6		4.8	
High school	3.29 (0.04)		37.2		17.1		6.7		4.1	
Sexual intercourse after drinking										
No	3.27 (0.03)	2.98 (0.084)	34.2	10.51 (0.001)	16.1	6.85 (0.013)	5.1	20.19 (<0.001)	3.8	21.53 (<0.001)
Yes	3.36 (0.04)		42.5		21.5		11.3		9.6	
Contraception use										
No	3.32 (0.06)	1.66 (0.174)	40.1	7.44 (0.118)	20.5	4.69 (0.206)	9.6	16.43 (0.005)	8.7	18.56 (0.003)
Sometimes	3.44 (0.08)		42.4		18.7		12.2		9.4	
Mostly	3.23 (0.07)		37.8		19.5		6.7		4.3	
Always	3.27 (0.04)		33.8		15.8		4.8		3.6	
Contraception method ^‡^										
Oral contraceptive pill	3.11 (0.15)	0.73 (0.571)	35.7	11.03 (0.027)	17.3	10.19 (0.058)	4.0	38.22 (<0.001)	4.0	30.54 (<0.001)
Condom	3.30 (0.04)		35.6		16.1		5.4		4.2	
Extravaginal ejaculation	3.32 (0.11)		31.6		18.9		5.6		1.9	
Others ^††^	3.03 (0.23)		59.5		35.7		39.8		26.7	
Sex education in school ^§^										
No	3.32 (0.06)	0.15 (0.703)	36.5	0.10 (0.738)	17.5	0.14 (0.706)	9.2	3.44 (0.075)	7.8	4.45 (0.070)
Yes	3.29 (0.03)		37.4		18.3		6.5		5.0	

* Weighted; ^†^ excluded missing values; ^‡^ among respondents who have ever used contraception; ^††^ This category included menstrual cycle method, emergency contraceptive pill, and intrauterine device; ^§^ in the last year.

**Table 3:** Differences in mental health according to sexual behavior in girls (*n* = 871).

Variables	Perceived Stress		Depression		Suicidal Ideation		Suicidal Plans		Suicidal Attempts	
	M * (SE)	Wald F (*p*)	% *	Rao-Scott χ^2^ (*p*)	% *	Rao-Scott χ^2^ (*p*)	% *	Rao-Scott χ^2^ (*p*)	% *	Rao-Scott χ^2^ (*p*)
Sexual debut time ^†^										
Elementary school or before	3.51 (0.18)	1.83 (0.161)	50.9	0.70 (0.730)	40.3	5.34 (0.090)	30.5	15.30 (<0.001)	27.7	15.72 (<0.001)
Middle school	3.82 (0.05)		55.6		26.8		12.5		12.1	
High school	3.73 (0.04)		53.2		27.5		12.0		9.7	
Sexual intercourse after drinking										
No	3.72 (0.04)	0.49 (0.481)	52.1	1.33 (0.223)	26.2	2.66 (0.115)	13.0	0.90 (0.345)	10.2	4.20 (0.033)
Yes	3.77 (0.05)		56.2		31.4		15.3		14.9	
Contraception use										
No	3.72 (0.08)	1.03 (0.380)	53.0	5.55 (0.148)	31.6	3.74 (0.305)	16.9	6.98 (0.068)	16.9	10.62 (0.013)
Sometimes	3.73 (0.09)		55.4		27.5		13.8		5.7	
Mostly	3.82 (0.06)		61.1		31.8		18.1		14.6	
Always	3.71 (0.04)		50.5		25.4		10.9		10.6	
Contraception method ^‡^										
Oral contraceptive pill	3.60 (0.15)	1.95 (0.100)	41.3	3.49 (0.569)	26.9	1.64 (0.775)	9.9	3.23 (0.510)	7.6	5.67 (0.198)
Condom	3.77 (0.03)		54.4		27.8		12.6		11.0	
Extravaginal ejaculation	3.83 (0.12)		52.9		25.3		15.1		11.7	
Others ^††^	3.11 (0.25)		63.8		25.0		19.3		6.7	
Sex education in school ^§^										
No	3.83 (0.07)	2.32 (0.128)	49.9	1.446 (0.177)	23.2	3.14 (0.077)	12.4	0.47 (0.475)	11.7	0.01 (0.905)
Yes	3.71 (0.04)		54.7		29.6		14.3		12.1	
Pregnancy experience ^||^										
No	3.78 (0.03)	7.47 (0.007)	53.2	1.47 (0.231)	26.8	9.90 (0.003)	12.3	17.25 (<0.001)	10.3	23.11 (<0.001)
Yes	3.21 (0.20)		61.3		46.8		34.2		35.1	

* Weighted; ^†^ excluded missing values; ^‡^ among respondents who have ever used contraception; ^††^ This category included menstrual cycle method, emergency contraceptive pill, and intrauterine device; ^§^ in the last year; ^||^ female students only.

## Data Availability

Not applicable.
